# Supplementary figures and images for: Amianthoid transformation of costal cartilage matrix in children with pectus excavatum and pectus carinatum
Source: PLoS One. 2021 Jan 25;16(1):e0245159. doi: 10.1371/journal.pone.0245159 (PMC7833175; doi:10.1371/journal.pone.0245159)

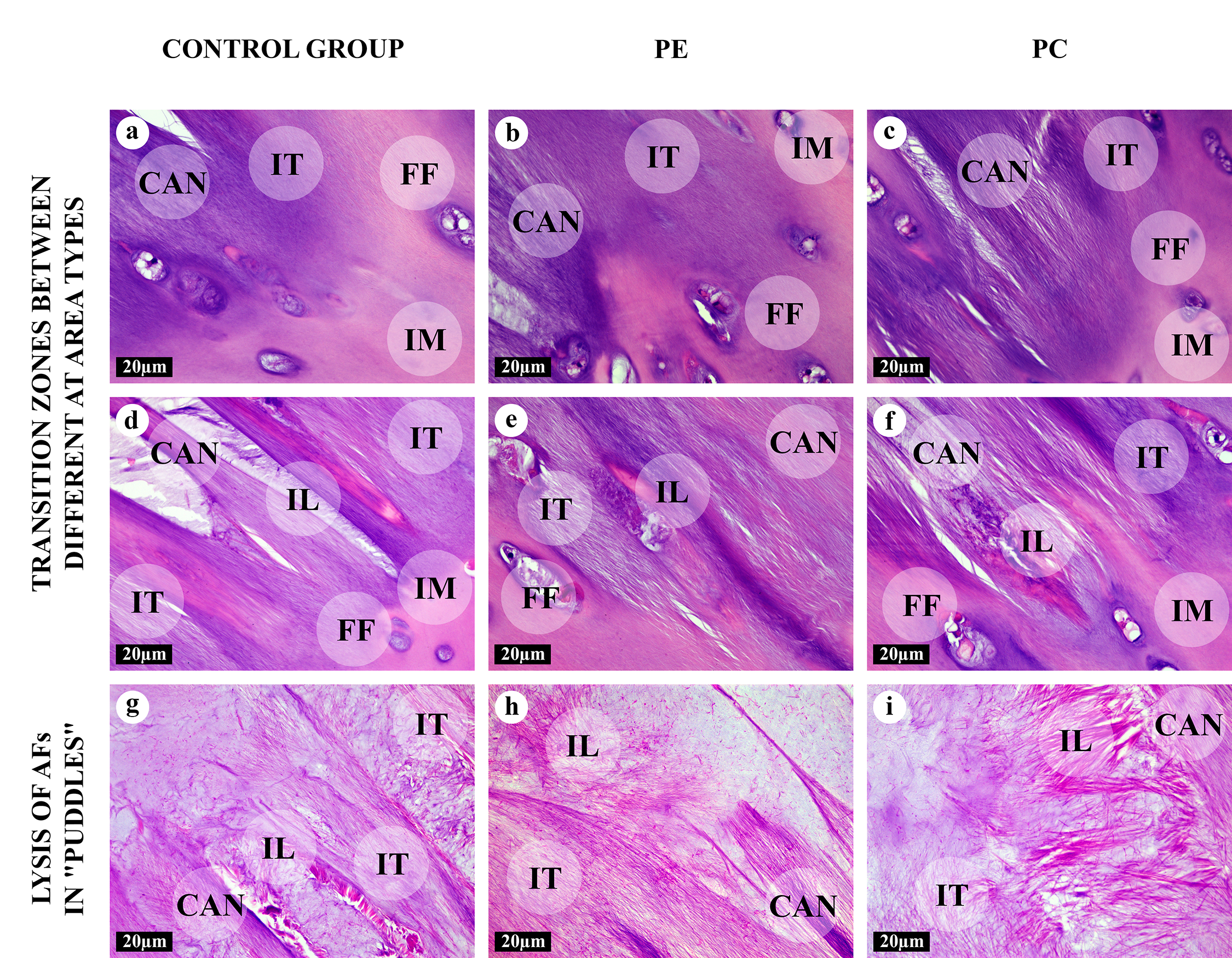

Supplement: S1 Fig — Often, different types of matrix did not have clear boundaries and mixed with each other, forming intermediate, or transitional, forms. The "canonical" (CAN) and "intertwined" (IT) AT areas were separated from the intact matrix (IM) by the "fine-fibred" (FF) AT area. Upon the destruction of the capsule of degenerating cartilaginous lacunae in AT areas of the "canonical" and "intertwined" types, the contents of the lacunae merged with the surrounding matrix. "Intralacunary" (IL) AFs merged with the AFs of these two types of the AT. Amorphous basophilic contents of lacunae, cellular detritus, were observed as “puddles” in the AT areas of the “canonical” and “intertwined” types. These "puddles" could contain the foci of lysis of the "canonical", "intertwined" or "intralacunary" AFs, as well as cartilaginous lacunae. Such changes did not occur in the intact matrix or in the "fine-fibred" AT areas. (TIF) [file pone.0245159.s001.tif]

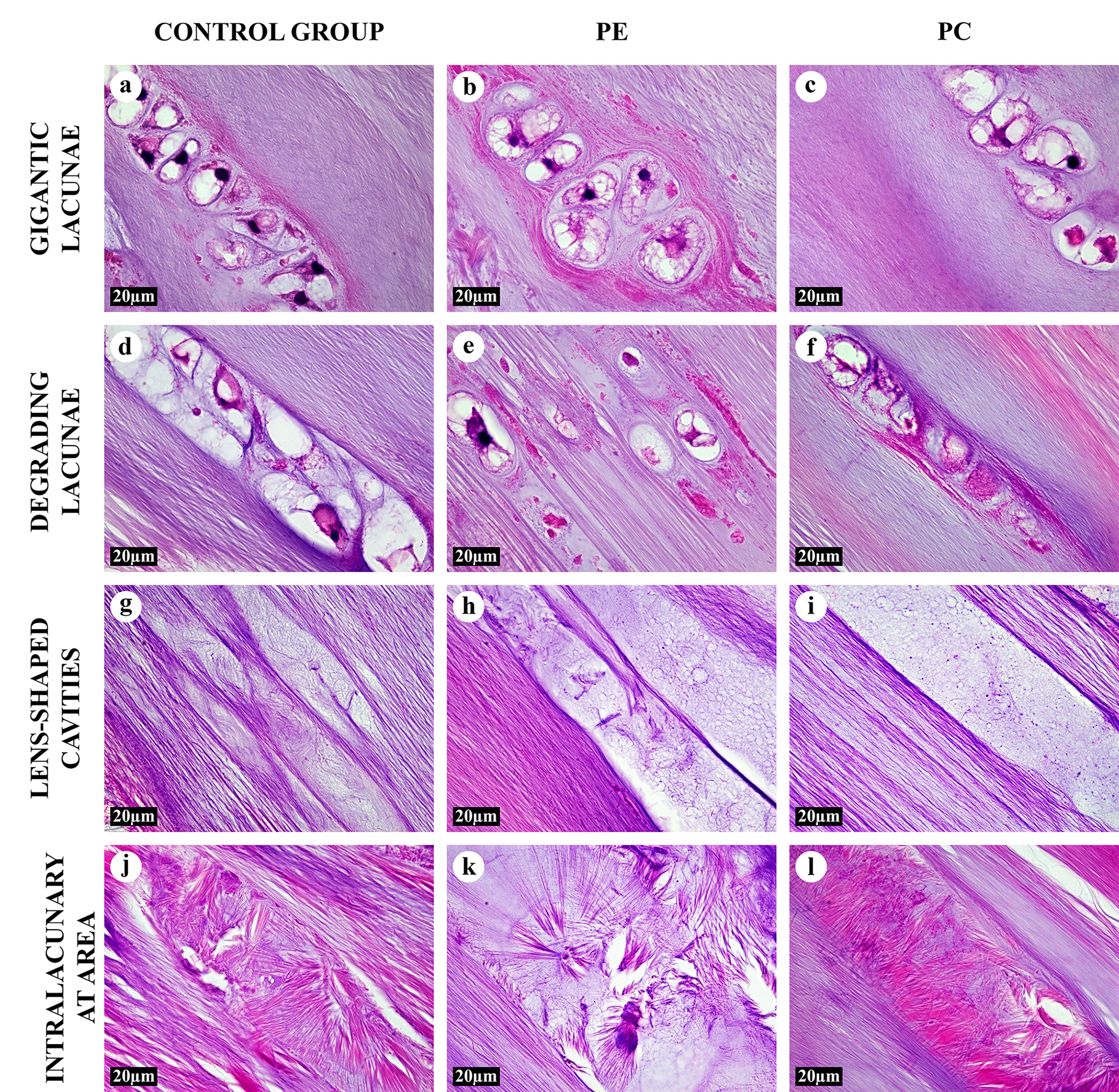

Supplement: S2 Fig — Chondrocytes were in a pronounced dystrophic state combined with hyperplasia and the formation of giant lacunae surrounded by a capsule. The destruction of chondrocytes resulted in formation of lens-shaped lacunae filled with homogeneous basophilic contents, cellular detritus, or "intralacunary" AFs. (TIF) [file pone.0245159.s002.tif]

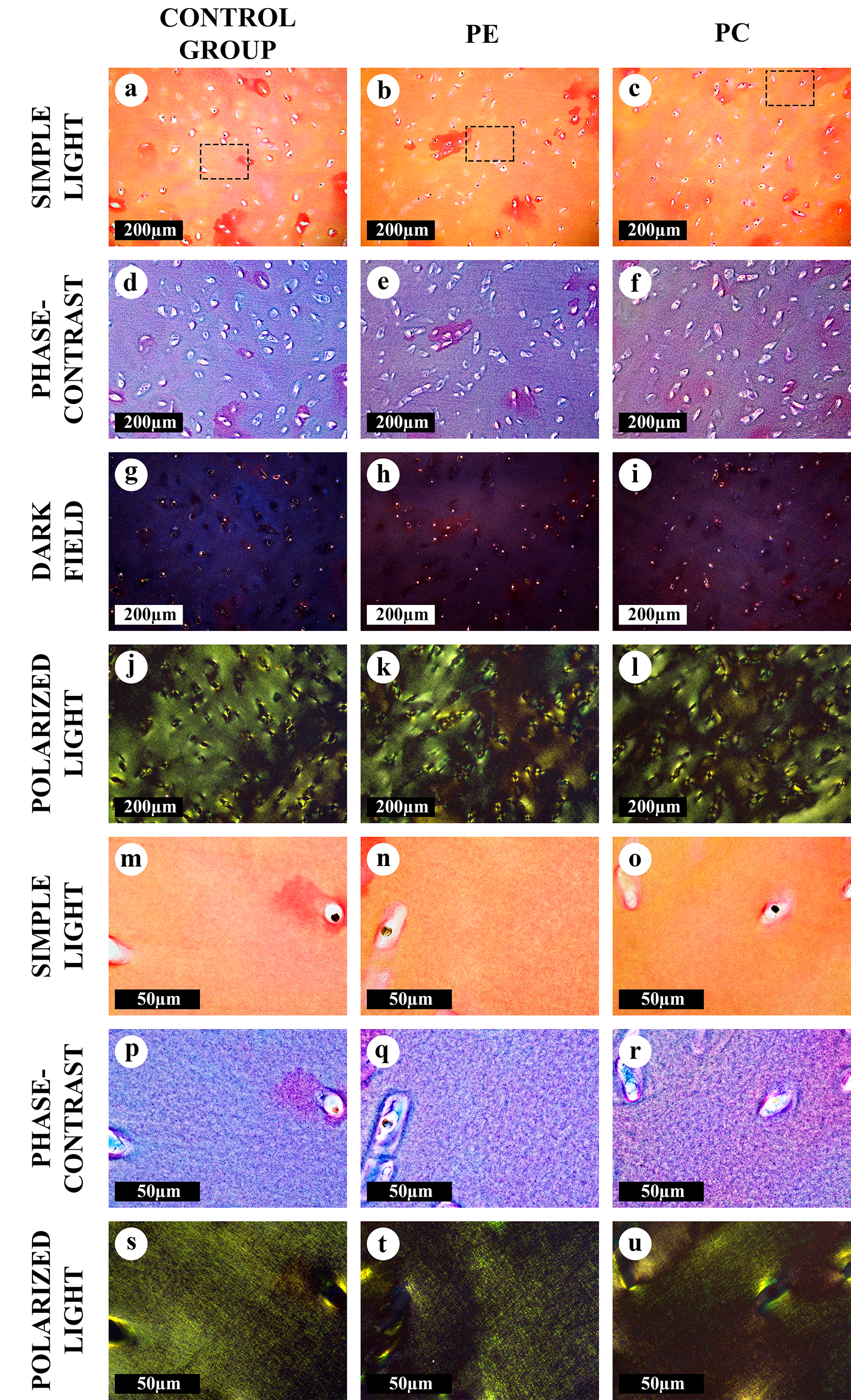

Supplement: S3 Fig — The intact matrix consisted of barely visible thin fibrils with anisotropic properties. (TIF) [file pone.0245159.s003.tif]

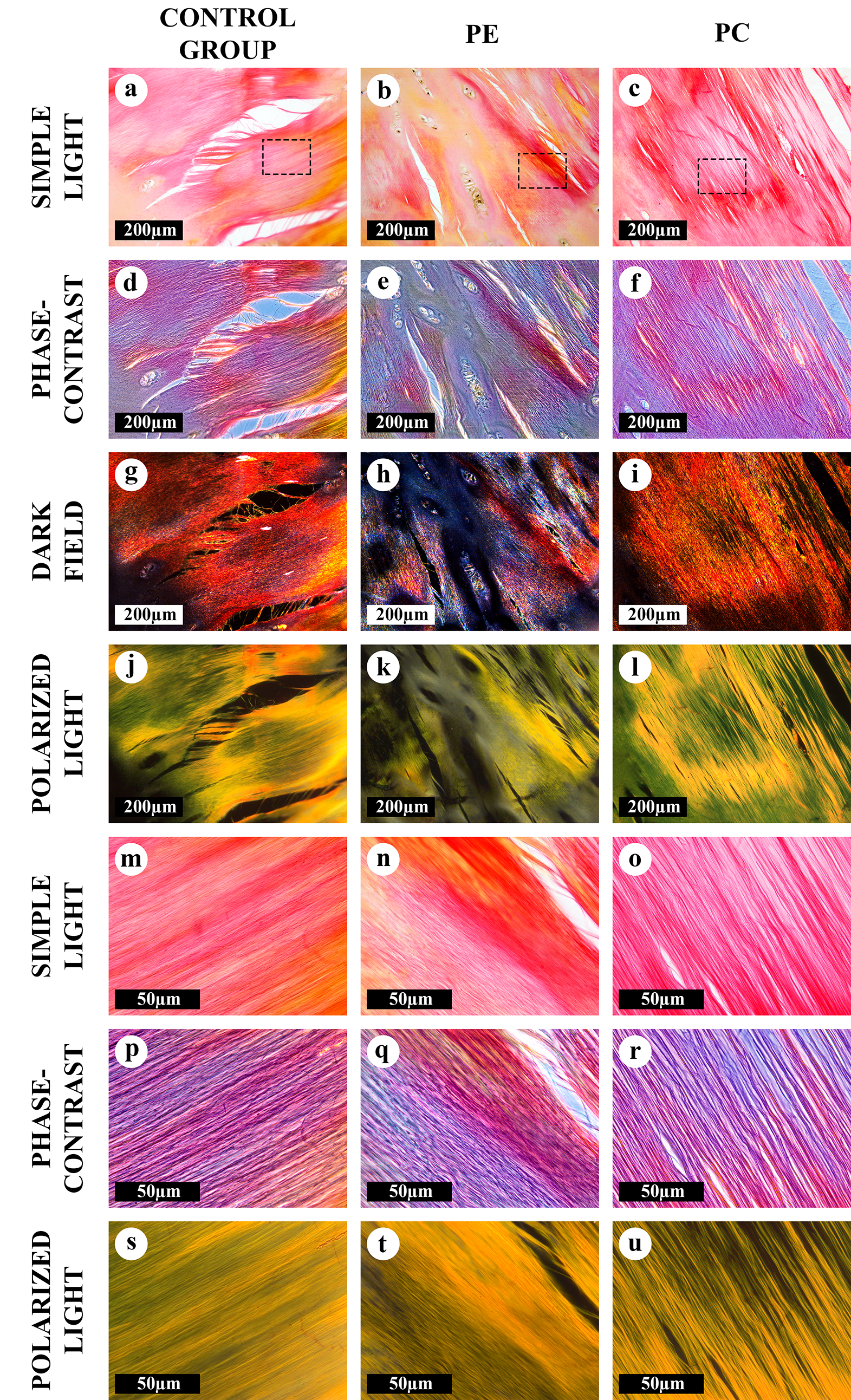

Supplement: S4 Fig — AT areas had a distinct fibrillar structure seen by phase-contrast microscopy. The AFs were brighter than thin fibrils of the intact matrix under dark-field and polarized light microscopies. (TIF) [file pone.0245159.s004.tif]

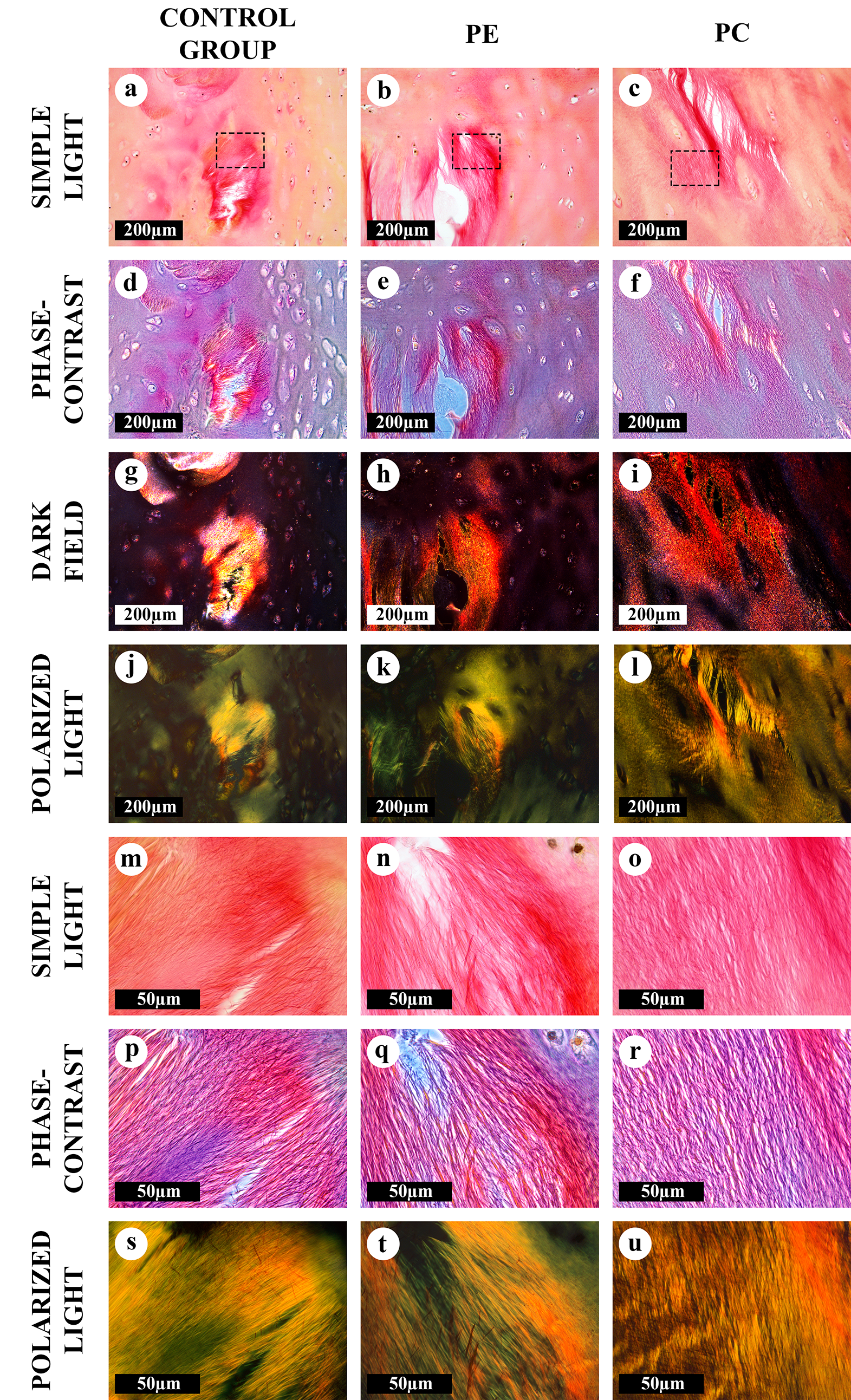

Supplement: S5 Fig — AT areas had a distinct fibrillar structure seen by phase-contrast microscopy. The AFs were brighter than thin fibrils of the intact matrix under dark-field and polarized light microscopies. (TIF) [file pone.0245159.s005.tif]

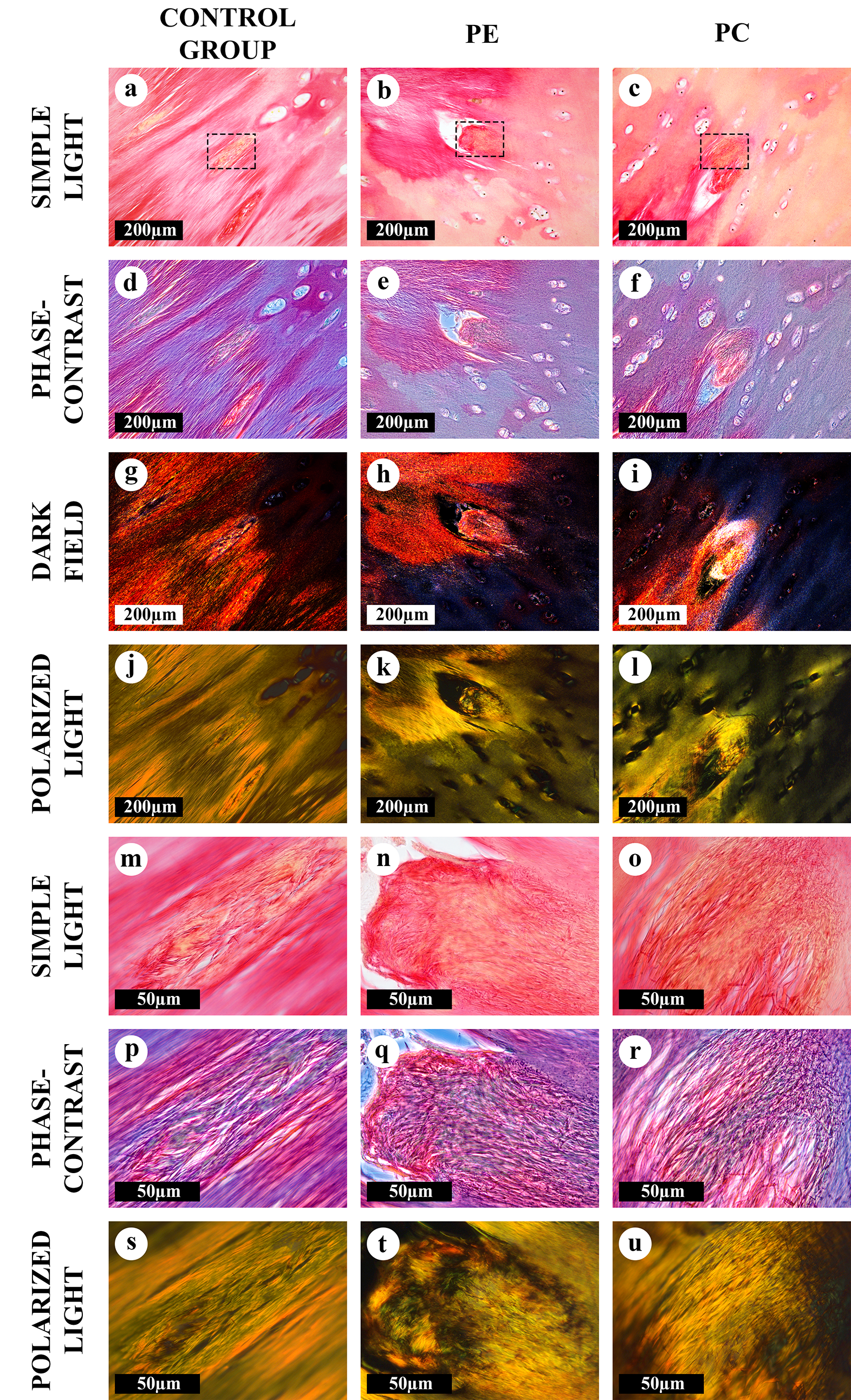

Supplement: S6 Fig — AT areas had a distinct fibrillar structure seen by phase-contrast microscopy. The AFs were brighter than thin fibrils of the intact matrix under dark-field and polarized light microscopies. (TIF) [file pone.0245159.s006.tif]

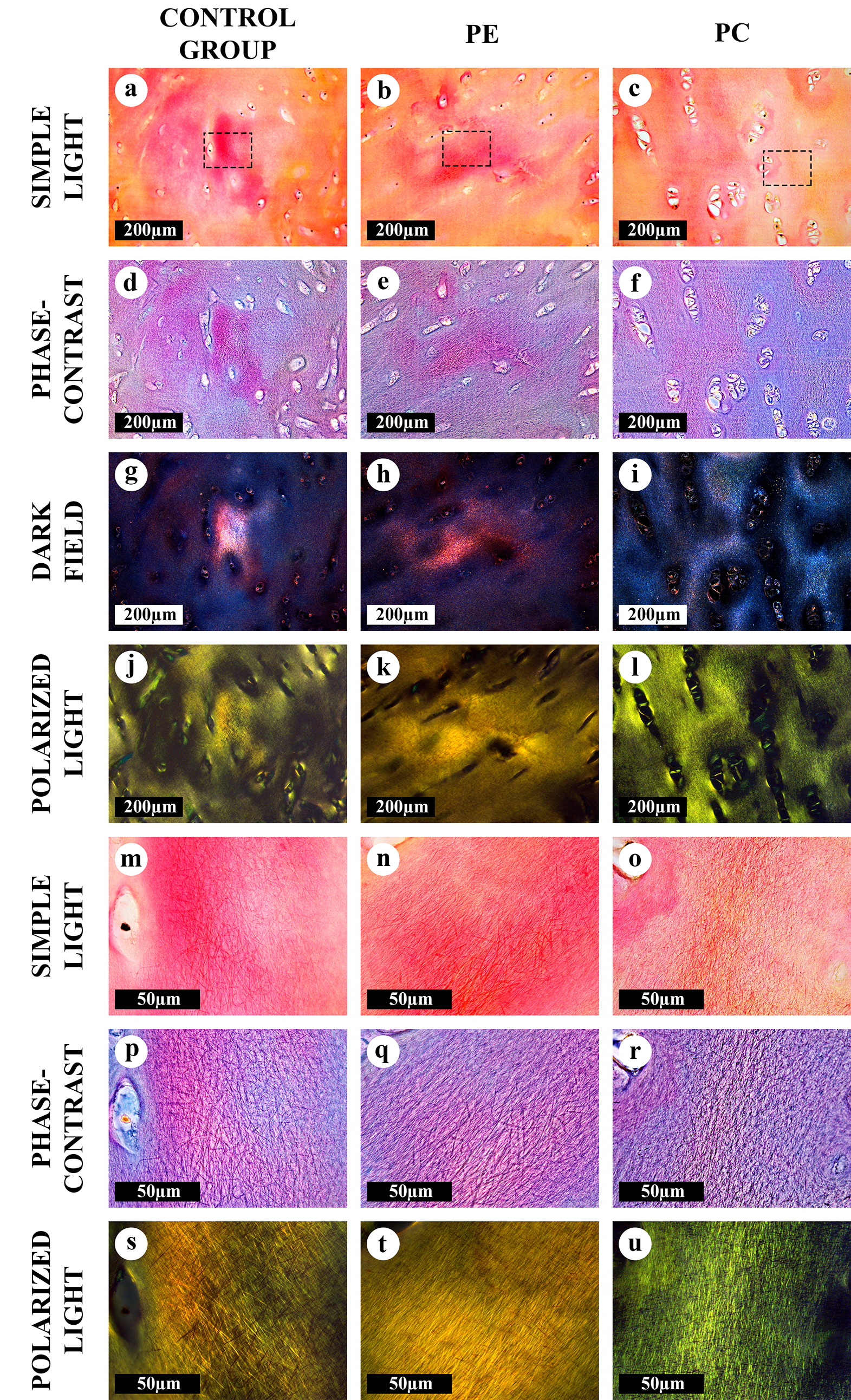

Supplement: S7 Fig — AT areas had a distinct fibrillar structure seen by phase-contrast microscopy. The AFs were brighter than thin fibrils of the intact matrix under dark-field and polarized light microscopies. (TIF) [file pone.0245159.s007.tif]

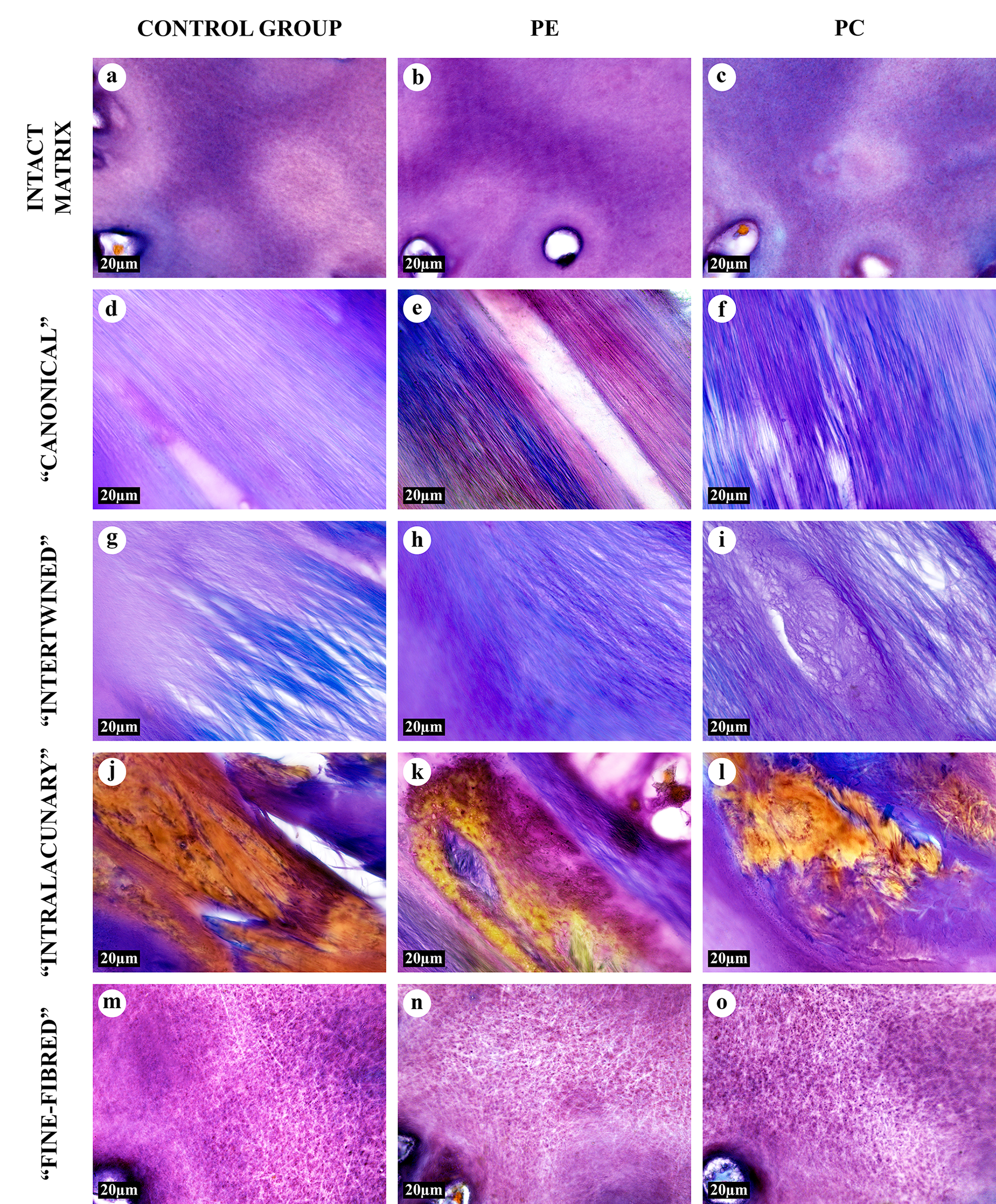

Supplement: S8 Fig — Tinctorial properties of the intact matrix (A-C) and four types of AT areas (D-O), stained by Mallory method, ×1000. AFs in “fine-fibred” AT areas (M-O) were colorless as compared to gray-blue-purple intact matrix (A-C) and blue AFs in other types of AT (D-L). Degenerating lacunae with “intralacunary” type AF contained orange-yellow detritus (J-L). (TIF) [file pone.0245159.s008.tif]

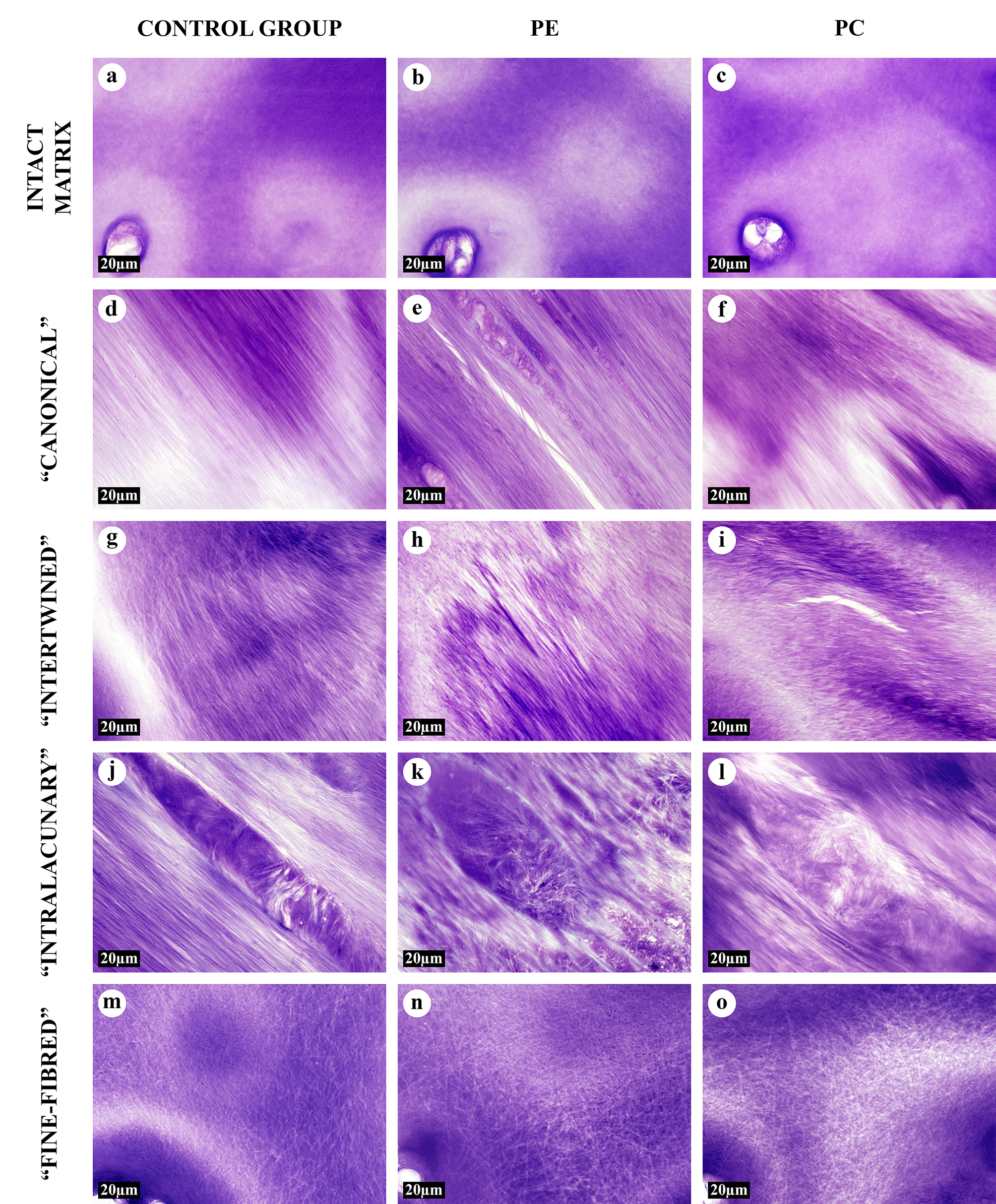

Supplement: S9 Fig — Tinctorial properties of the intact matrix (A-C) and four types of AT areas (D-O), stained with toluidine blue, ×1000. AFs in “fine-fibred” AT areas (M-O) were colorless as compared to blue-purple intact matrix (A-C). AFs in other types of AT (D-L) demonstrated uneven metachromasia. (TIF) [file pone.0245159.s009.tif]

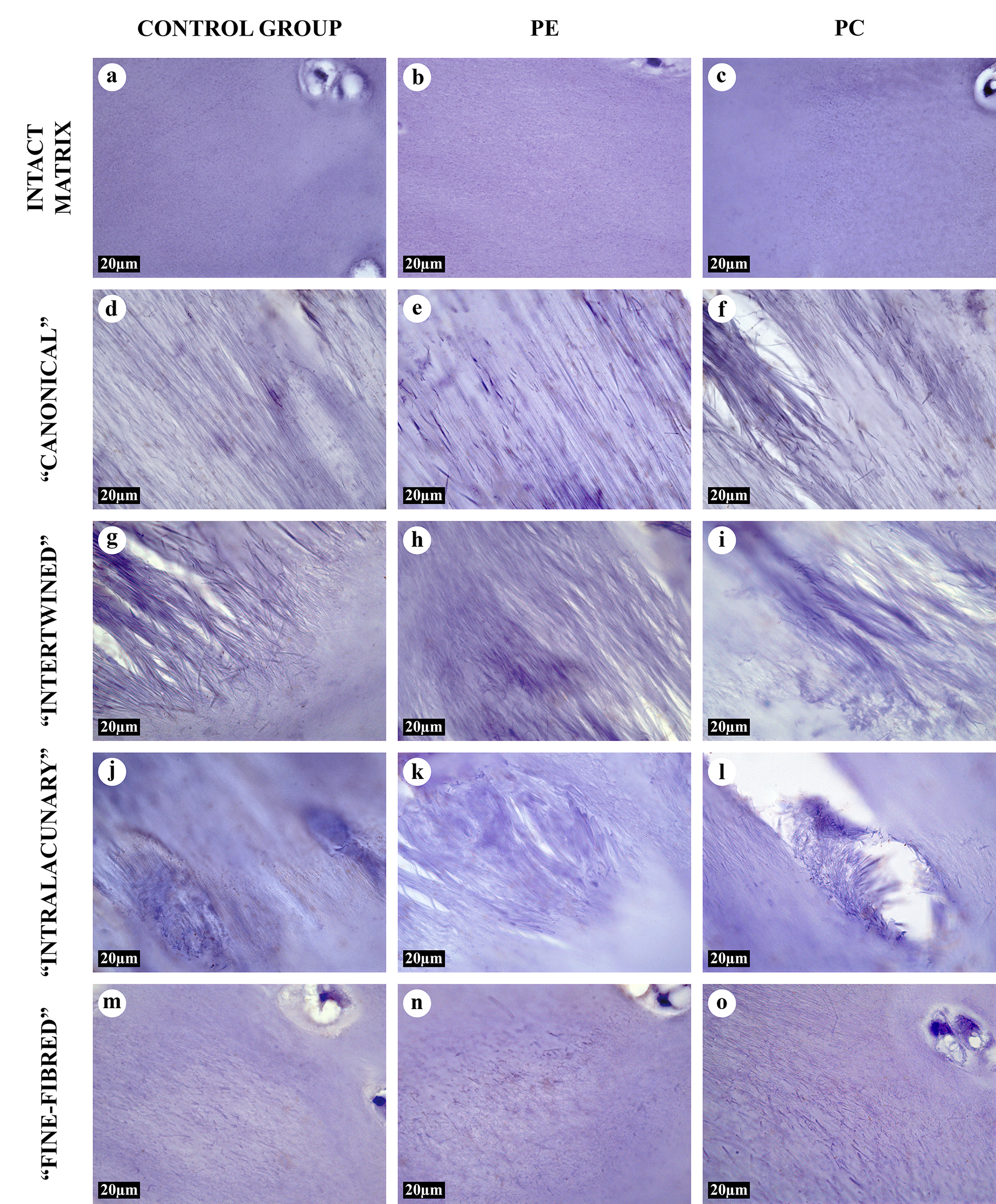

Supplement: S10 Fig — Collagen type I expression was absent in chondrocytes and all matrix types. (TIF) [file pone.0245159.s010.tif]

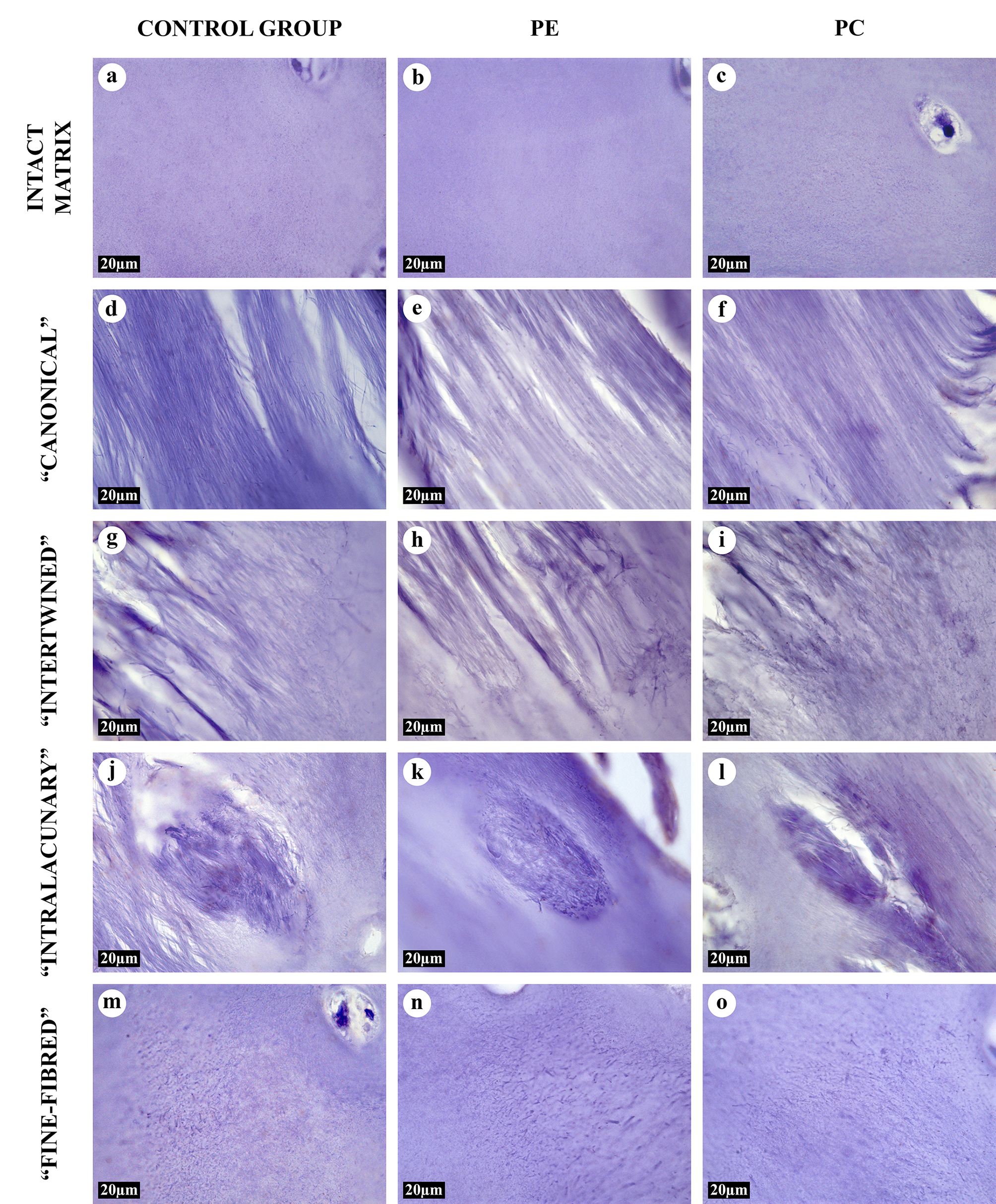

Supplement: S11 Fig — Collagen type III expression was absent in chondrocytes and all matrix types. (TIF) [file pone.0245159.s011.tif]

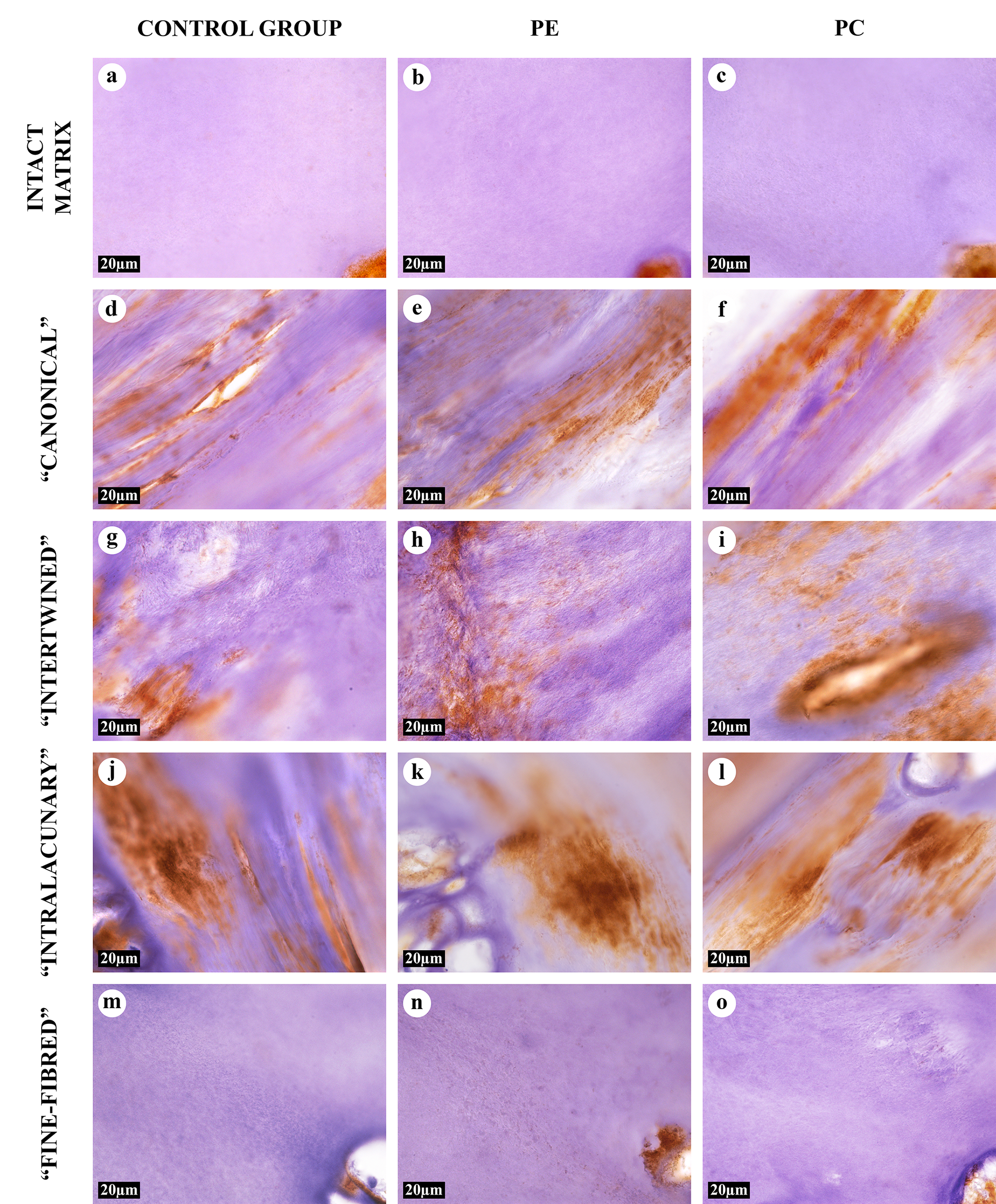

Supplement: S12 Fig — Collagen type II expression was revealed in chondrocytes (positive internal control). Intact matrix and "fine-fibred" AT areas were not stained. The matrix of other types of AT areas had weak, uneven staining. However, most AFs did not stain positively. (TIF) [file pone.0245159.s012.tif]

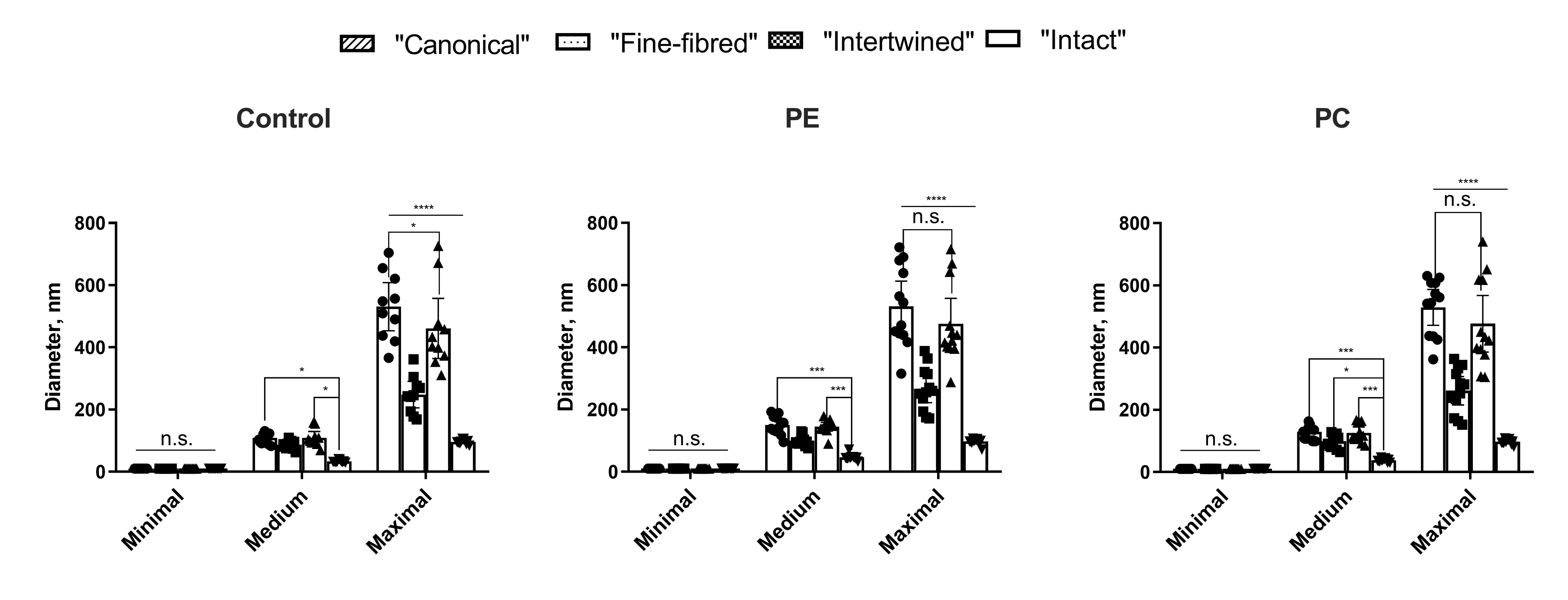

Supplement: S13 Fig — Statistical significance: **—p ≤ 0.01, ****—p ≤ 0.0001, n.s.–non-significant. Two-way ANOVA followed by the Tukey's test, interleaved scatter with bars, mean values ± 95% confidence intervals. (TIF) [file pone.0245159.s013.tif]
