# Supplementary material for: Amianthoid transformation of costal cartilage matrix in children with pectus excavatum and pectus carinatum
Source: PLoS One. 2021 Jan 25;16(1):e0245159. doi: 10.1371/journal.pone.0245159 (PMC7833175; doi:10.1371/journal.pone.0245159)
Supplement: S1 Table — (DOCX) [file pone.0245159.s014.docx]

**S1 Table. Correlation analysis (only significant correlations are shown).**

| Control | Pectus carinatum | Pectus excavatum |
| --- | --- | --- |
| Correlation coefficients between the patients’ age and total area of AT foci: | | |
| - all the types (in total): +0.467 (p=0.001); - “canonical” type: +0.868   (p<0.001);   - “intralacunary” type: +0.557 (p=0.005); - “intertwined” type: +0.422 (p=0.04); | - “intralacunary” type: - +0.576 (p=0.031); | - “intralacunary” type: +0.652 (p=0.008); - “intertwined” type:   +0.644 (p=0.01); |
| Correlation coefficients between areas of different types of AT foci: | | |
| - between the “intertwined” and “canonical” types: +0.48 (p=0.018); - between the “intralacunary” and “canonical” types: +0.594 (p=0.002); - between the “intralacunary” and “intertwined” types:   +0.513 (p=0.01); | - between the “fine-fibred” and “canonical” types: -0.670 (p=0.009); | - between the “fine-fibred” and “canonical” types: -0.576 (p=0.016), - between the   “intertwined” and “fine-fibred” types: -0.554  (p=0.021). |
